# Supplementary material for: Discrimination of Bacillus cereus Group Members by MALDI-TOF Mass Spectrometry
Source: Microorganisms. 2021 Jun 2;9(6):1202. doi: 10.3390/microorganisms9061202 (PMC8228078; doi:10.3390/microorganisms9061202)
Supplement: Supplementary file 1 [file microorganisms-09-01202-s001.zip › Table S1.pdf]

Table S1. List of *Bacillus cereus* group strains used in this study.

Code (ID)	Origin (Source)	Bacterial strains
BA002	Sheep (anthrax outbreak in Apulia region of 1993)	*B. anthracis (pXO1$^+$/pXO2$^+$)*
BA004	Goat (anthrax outbreak in Apulia region of 1993)	*B. anthracis (pXO1$^+$/pXO2$^+$)*
BA005	Sheep (anthrax outbreak in Apulia region of 1993)	*B. anthracis (pXO1$^+$/pXO2$^+$)*
BA008	Sheep (anthrax outbreak in Sicily region of 1996)	*B. anthracis (pXO1$^+$/pXO2$^+$)*
BA010	Donkey (anthrax outbreak in Sicily region of 1996)	*B. anthracis (pXO1$^+$/pXO2$^+$)*
BA019	Cattle (anthrax outbreak in Basilicata region of 1999)	*B. anthracis (pXO1$^+$/pXO2$^+$)*
BA021	34F2 Sterne vaccine strain	*B. anthracis (pXO1$^+$/pXO2$^-$)*
BA022	Sheep (anthrax outbreak in Apulia region of 1984)	*B. anthracis (pXO1$^+$/pXO2$^+$)*
BA031	Sheep (anthrax outbreak in Apulia region of 1984)	*B. anthracis (pXO1$^+$/pXO2$^+$)*
BA045	Sheep (anthrax outbreak in Apulia region of 2000)	*B. anthracis (pXO1$^+$/pXO2$^+$)*
BA048	Cattle (anthrax outbreak in Sardinia region of 2000)	*B. anthracis (pXO1$^+$/pXO2$^+$)*
BA049	Cattle (anthrax outbreak in Veneto region of 1992)	*B. anthracis (pXO1$^+$/pXO2$^+$)*
BA051	Human (anthrax outbreak in Lazio region of 1999)	*B. anthracis (pXO1$^+$/pXO2$^+$)*
BA052	Sheep (anthrax outbreak in Lazio region of 1999)	*B. anthracis (pXO1$^+$/pXO2$^+$)*
BA053	Cattle (anthrax outbreak in Tuscany region of 1999)	*B. anthracis (pXO1$^+$/pXO2$^+$)*
BA058	Sheep (anthrax outbreak in Apulia region of 2001)	*B. anthracis (pXO1$^+$/pXO2$^+$)*
BA063	Sheep (anthrax outbreak in Basilicata region of 2002)	*B. anthracis (pXO1$^+$/pXO2$^+$)*
BA065	Goat (anthrax outbreak in Calabria region of 2002)	*B. anthracis (pXO1$^+$/pXO2$^+$)*
BA103	Deer (anthrax outbreak in Basilicata region of 2004)	*B. anthracis (pXO1$^+$/pXO2$^+$)*
BA105	Soil (cursed fields in Basilicata region -2004)	*B. anthracis (pXO1$^+$/pXO2$^+$)*
BA120	Swine (anthrax outbreak in Basilicata region of 2004)	*B. anthracis (pXO1$^+$/pXO2$^+$)*
BA124	Goat (anthrax outbreak in Sardinia region of 1996)	*B. anthracis (pXO1$^+$/pXO2$^+$)*
BA132	Goat (anthrax outbreak in Apulia region of 2005)	*B. anthracis (pXO1$^+$/pXO2$^+$)*
BA135	Cattle (anthrax outbreak in Sicily region of 2005)	*B. anthracis (pXO1$^+$/pXO2$^+$)*
BA140	Cattle (anthrax outbreak in Basilicata region of 2006)	*B. anthracis (pXO1$^+$/pXO2$^+$)*
BA145	Cattle (anthrax outbreak in Tuscany region of 2008)	*B. anthracis (pXO1$^+$/pXO2$^+$)*
BA152	Cattle (anthrax outbreak in Umbria region of 2009)	*B. anthracis (pXO1$^+$/pXO2$^+$)*
BA156	Soil (cursed fields in Tuscany region -2009)	*B. anthracis (pXO1$^+$/pXO2$^+$)*
BA174	Cattle (anthrax outbreak in Sicily region of 2009)	*B. anthracis (pXO1$^+$/pXO2$^+$)*
BA175	Sheep (anthrax outbreak in Basilicata region of 2009)	*B. anthracis (pXO1$^+$/pXO2$^+$)*
BA180	Cattle (anthrax outbreak in Sicily region of 2009)	*B. anthracis (pXO1$^+$/pXO2$^+$)*
BA181	Sheep (anthrax outbreak in Campania region of 2009)	*B. anthracis (pXO1$^+$/pXO2$^+$)*
BA182	Cattle (anthrax outbreak in Basilicata region of 2009)	*B. anthracis (pXO1$^+$/pXO2$^+$)*
BA183	Cattle (anthrax outbreak in Lombardia region of 1989)	*B. anthracis (pXO1$^+$/pXO2$^+$)*
BA184	Cattle (anthrax outbreak in Apulia region of 2009)	*B. anthracis (pXO1$^+$/pXO2$^+$)*
BA187	Cattle (anthrax outbreak in Trentino Alto Adige region of 2006)	*B. anthracis (pXO1$^+$/pXO2$^+$)*
BA188	Cattle (anthrax outbreak in Veneto region of 2007)	*B. anthracis (pXO1$^+$/pXO2$^+$)*
BA300	Equine (anthrax outbreak in Campania region of 2011)	*B. anthracis (pXO1$^+$/pXO2$^+$)*
BA308	Sheep (anthrax outbreak in Basilicata region of 2011)	*B. anthracis (pXO1$^+$/pXO2$^+$)*
BA421	Cattle (anthrax outbreak in Sicily region of 2012)	*B. anthracis (pXO1$^+$/pXO2$^+$)*
BA430	Human (anthrax outbreak in Basilicata region of 2012)	*B. anthracis (pXO1$^+$/pXO2$^+$)*
BA434	Soil (cursed fields in Basilicata region - 2012)	*B. anthracis (pXO1$^+$/pXO2$^+$)*
BA439	Cattle (anthrax outbreak in Sicily region of 2012)	*B. anthracis (pXO1$^+$/pXO2$^+$)*
BA441	Cattle (anthrax outbreak in Tuscany region of 2012)	*B. anthracis (pXO1$^+$/pXO2$^+$)*

BA651	Soil (cursed fields in Basilicata region - 2014)	*B. anthracis (pXO1$^+$/pXO2$^+$)*
BA661	Cattle (anthrax outbreak in Basilicata region of 2014)	*B. anthracis (pXO1$^+$/pXO2$^+$)*
BA662	Cattle (anthrax outbreak in Sicily region of 2014)	*B. anthracis (pXO1$^+$/pXO2$^+$)*
BA718	Goat (anthrax outbreak in Abruzzo region of 2016)	*B. anthracis (pXO1$^+$/pXO2$^+$)*
BA719	Sheep (anthrax outbreak in Lazio region of 2016)	*B. anthracis (pXO1$^+$/pXO2$^+$)*
BA722	Cattle (anthrax outbreak in Sicily region of 2016)	*B. anthracis (pXO1$^+$/pXO2$^+$)*
BA726	Cattle (anthrax outbreak in Lazio region of 2017)	*B. anthracis (pXO1$^+$/pXO2$^+$)*
BA729	Sheep (anthrax outbreak in Campania region of 2017)	*B. anthracis (pXO1$^+$/pXO2$^+$)*
BA730	Cattle (anthrax outbreak in Lazio region of 2018)	*B. anthracis (pXO1$^+$/pXO2$^+$)*
BA733	Cattle (anthrax outbreak in Sicily region of 2018)	*B. anthracis (pXO1$^+$/pXO2$^+$)*
BA741	Cattle (anthrax outbreak in Apulia region of 2019)	*B. anthracis (pXO1$^+$/pXO2$^+$)*
BA742	Cattle (anthrax outbreak in Calabria region of 2020)	*B. anthracis (pXO1$^+$/pXO2$^+$)*
BA743	Goat (anthrax outbreak in Campania region of 2020)	*B. anthracis (pXO1$^+$/pXO2$^+$)*
BCG126	Mozzarella	*B. cereus*
BCG128	Mozzarella	*B. cereus*
BCG129	Mozzarella	*B. cereus*
BCG131	Mozzarella	*B. cereus*
BCG132	Buffalo mozzarella	*B. cereus*
BCG134	Burrata	*B. cereus*
BCG135	Raw cow's milk	*B. cereus*
BCG136	Raw cow's milk	*B. cereus*
BCG137	Pasteurized milk	*B. cereus*
BCG139	Mozzarella	*B. cereus*
BCG145	Bovine meat	*B. cereus*
BCG146	Butter	*B. cereus*
BCG148	Mozzarella	*B. cereus*
BCG149	Mozzarella	*B. cereus*
BCG150	Mozzarella	*B. cereus*
BCG152	Yogurt	*B. cereus*
BCG153	Wurstel	*B. cereus*
BCG157	Fresh cheese	*B. cereus*
BCG164	Swordfish with olives	*B. cereus*
BCG165	Butter	*B. cereus*
BCG169	Common wheat flour	*B. cereus*
BCG171	Mozzarella	*B. cereus*
BCG174	Egg pasta (pappardelle)	*B. cereus*
BCG178	Bulk milk	*B. cereus*
BCG180	Ice cream	*B. cereus*
BCG183	Goat cheese	*B. cereus*
BCG184	Rocket salad	*B. cereus*
BCG191	Mozzarella	*B. cereus*
BCG192	Caciocavallo	*B. cereus*
BCG194	Caciocavallo	*B. cereus*
BCG197	Mozzarella	*B. cereus*
BCG198	Mozzarella	*B. cereus*
BCG199	Mozzarella	*B. cereus*
BCG200	Aged cheese	*B. cereus*
BCG201	Ricotta	*B. cereus*
BCG218	Pasta filata cheese	*B. cereus*

BCG222	Scamorza	*B. cereus*
BCG235	Artichokes	*B. cereus*
BCG243	Salad	*B. cereus*
BCG249	Packaged ice cream cone	*B. cereus*
BCG250	Industrial biscuits	*B. cereus*
BCG251	Industrial biscuits	*B. cereus*
BCG253	Industrial biscuits	*B. cereus*
BCG254	Industrial biscuits	*B. cereus*
BCG255	Industrial chocolate biscuits	*B. cereus*
BCG263	Panettone	*B. cereus*
BCG265	Truffle	*B. cereus*
BCG294A	Mozzarella	*B. cereus*
BCG337A	Artichokes	*B. cereus*
BCG020	Food	*B. mycoides*
BCG140	Fresh sweet sausage	*B. thuringensis*
BCG142	Bulk milk	*B. thuringensis*
BCG144	Ice cream	*B. thuringensis*
BCG151	Fiordilatte	*B. thuringensis*
BCG155	Mozzarella	*B. thuringensis*
BCG156	Giuncata	*B. thuringensis*
BCG160	Smoked cheese	*B. thuringensis*
BCG168	Aged cheese	*B. thuringensis*
BCG172	Fresh cheese	*B. thuringensis*
BCG173	Mozzarella	*B. thuringensis*
BCG175	Focaccia with smoked cheese	*B. thuringensis*
BCG176	Ricotta	*B. thuringensis*
BCG179	Sandwich	*B. thuringensis*
BCG186	Caciotta	*B. thuringensis*
BCG188	Mozzarella	*B. thuringensis*
BCG189	Mozzarella	*B. thuringensis*
BCG190	Mozzarella	*B. thuringensis*
BCG202	Fava beans	*B. thuringensis*
BCG203	Finocchio	*B. thuringensis*
BCG204	Salad	*B. thuringensis*
BCG220	Caciocavallo	*B. thuringensis*
BCG224	Caciocavallo	*B. thuringensis*
BCG232	Chicory	*B. thuringensis*
BCG242	Green beans	*B. thuringensis*
BCG254A	Fiordilatte	*B. thuringensis*
BCG256	Industrial chocolate biscuits	*B. thuringensis*
BCG335A	Kale	*B. thuringensis*
BCG130	Mozzarella	*B. toyonensis*
BCG159	Mozzarella	*B. toyonensis*
BCG185	Mixed salad	*B. toyonensis*
BCG225	Mozzarella	*B. toyonensis*
BCG233	Rocket salad	*B. toyonensis*
BCG237	Salad	*B. toyonensis*
BCG262	Almond sweets	*B. toyonensis*
BCG147	Mozzarella	*B. weihenstephanensis*

BCG127	Mozzarella	*B. wiedmannii*
BCG133	Buffalo mozzarella	*B. wiedmannii*
BCG141	Caciocavallo	*B. wiedmannii*
BCG154	Mozzarella	*B. wiedmannii*
BCG161	Mozzarella	*B. wiedmannii*
BCG162	Mozzarella	*B. wiedmannii*
BCG167	Wurstel	*B. wiedmannii*
BCG177	Ricotta	*B. wiedmannii*
BCG182	Scamorza	*B. wiedmannii*
BCG187	Mozzarella	*B. wiedmannii*
BCG193	Mozzarella	*B. wiedmannii*
BCG195	Ricotta	*B. wiedmannii*
BCG226	Mozzarella	*B. wiedmannii*
BCG234	Salad	*B. wiedmannii*
BCG245	Egg pasta (ravioli)	*B. wiedmannii*
BCG248	Surimi	*B. wiedmannii*
BCG252	Wholemeal industrial biscuits	*B. wiedmannii*
BCG286A	Mozzarella	*B. wiedmannii*
