# Supplementary figures and images for: Discrimination of Bacillus cereus Group Members by MALDI-TOF Mass Spectrometry
Source: Microorganisms. 2021 Jun 2;9(6):1202. doi: 10.3390/microorganisms9061202 (PMC8228078; doi:10.3390/microorganisms9061202)

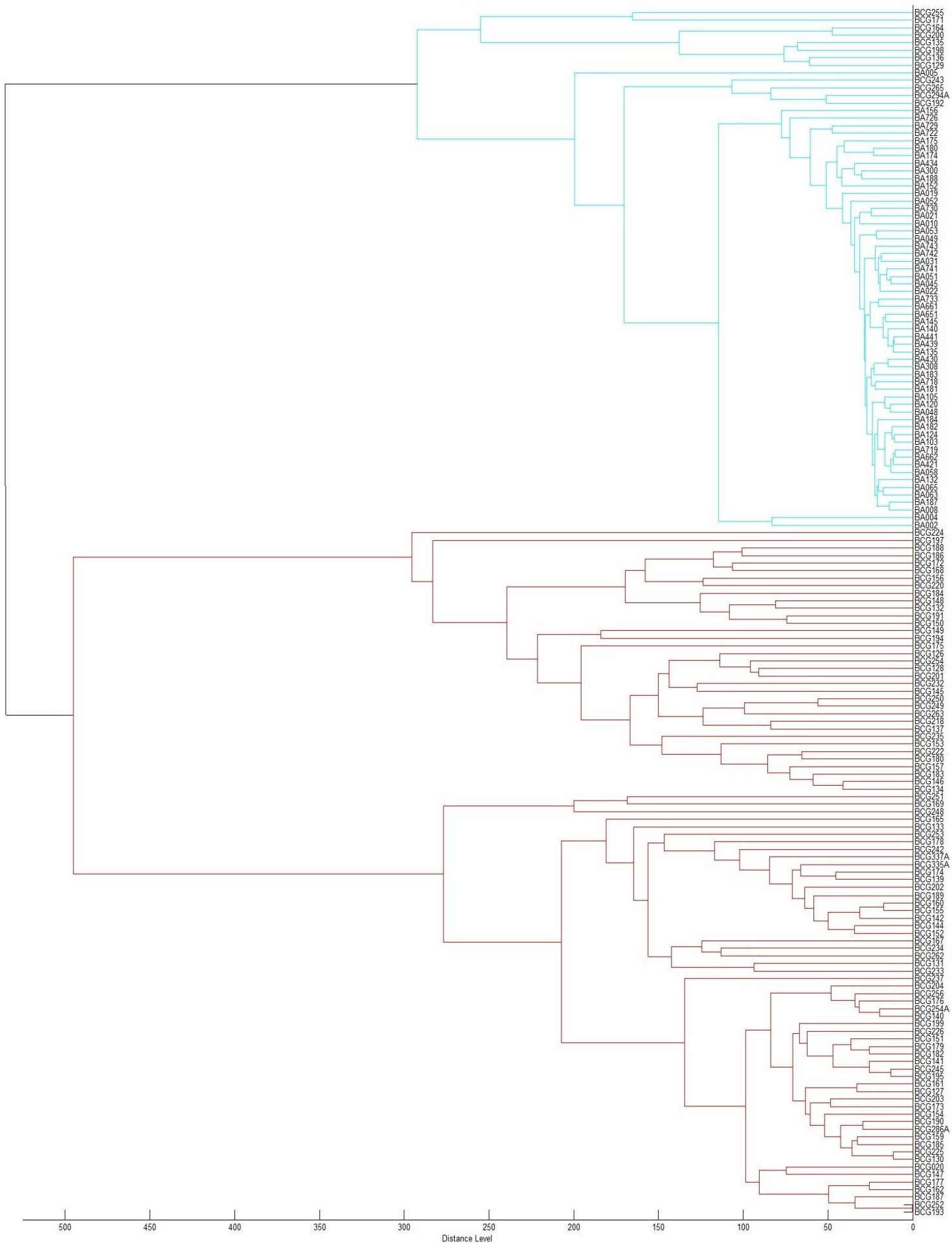

Supplement: Supplementary file 1 [file microorganisms-09-01202-s001.zip › Figure S1.pdf]
